# Open-Source, Low Cost, Free-Behavior Monitoring, and Reward System for Neuroscience Research in Non-human Primates

**DOI:** 10.3389/fnins.2017.00265

**Published:** 2017-05-16

**Authors:** Tyler Libey, Eberhard E. Fetz

**Affiliations:** ^1^Department of Bioengineering, University of WashingtonSeattle, WA, United States; ^2^Department of Physiology and Biophysics, University of WashingtonSeattle, WA, United States; ^3^National Science Foundation ERC Center for Sensorimotor Neural EngineeringSeattle, WA, United States

**Keywords:** brain machine interface, free behavior, operant conditioning, motion capture, wireless systems

## Abstract

We describe a low-cost system designed to document bodily movement and neural activity and deliver rewards to monkeys behaving freely in their home cage. An important application is to studying brain-machine interface (BMI) systems during free behavior, since brain signals associated with natural movement can differ significantly from those associated with more commonly used constrained conditions. Our approach allows for short-latency (<500 ms) reward delivery and behavior monitoring using low-cost off-the-shelf components. This system interfaces existing untethered recording equipment with a custom hub that controls a cage-mounted feeder. The behavior monitoring system uses a depth camera to provide real-time, easy-to-analyze, gross movement data streams. In a proof-of-concept experiment we demonstrate robust learning of neural activity using the system over 14 behavioral sessions.

## Introduction

Neuroscience research has often used non-human primates as the best animal model for behavioral and cognitive studies due to their similarities to humans (Anderson, 2008). Relationships between neural activity and behavior can be studied in controlled environments to elucidate, for example, how motor cortex neuron firing is related to muscle activity (Fetz and Finocchio, 1975) or how populations of neurons relate to complex reach and grasp movements (Vargas-Irwin et al., 2010). In these experiments the monkey typically sits in a specially designed chair or box used for restraint and transport. The head of the monkey is normally fixed to protect the recording equipment and reduce noise in the neural recordings. For studying behavior during neural recordings, experiments use mechanical systems such as joysticks (Ifft et al., 2012) or torque-tracking devices (Moritz and Fetz, 2011), implanted muscle activity recordings (Fetz and Finocchio, 1975; Griffin et al., 2008), or video monitoring systems (Chen et al., 2009; Vargas-Irwin et al., 2010). These systems offer the benefit of being heavily constrained, enabling precise documentation of controlled movements and tasks. However, these systems limit the animal's natural movement, restricting the types of movements that can be studied and therefore the real-world relevance of the results.

The relationship between neural activity and natural behavior is important for the development of brain-machine interfaces (BMIs). Many BMI studies have shown success in controlling external devices, such as computer cursors (Santhanam et al., 2006) and robotic arms (Taylor et al., 2002) using constrained monkeys. BMI systems have been implemented in humans, but they have been limited to patients suffering from tetraplegia (Hochberg et al., 2012; Wang et al., 2013) or during epilepsy treatments (Wander et al., 2013). These systems may not be ideal models for BMI applications during free behavior, as these populations do not exhibit full, natural motor movement. Studies have shown that the correlations between behavior and neural activity may differ when monkeys are behaving naturally in their home environment (Caminiti et al., 1990; Aflalo and Graziano, 2006; Jackson et al., 2007). To develop BMI systems that account for these differences we need to better understand the relationship between neural activity and natural behavior during BMI control.

Traditionally, neural signals are recorded in constrained animal via connecting wires to large, rack-mounted systems. Recently, wireless hardware systems have been designed to record neural signals while the monkey is freely behaving (Jackson et al., 2006b; Miranda et al., 2010; Zanos et al., 2011; Fernandez-Leon et al., 2015). These untethered systems can record multiple channels of high quality neural data with limited impact on the monkey's natural movement. The Neurochip system (Jackson et al., 2006b; Zanos et al., 2011) can record multiple channels of neural data, perform online computations, and provide electrical stimulation while a monkey is freely behaving in its cage. This battery powered system operates autonomously and stores data to a memory card. Untethered recording systems have been used to study the long-term effects of stimulation on neural plasticity (Jackson et al., 2006a), correlations between motor neurons and muscle activity during sleep (Jackson and Fetz, 2007), and the relationship between neural activity and untethered treadmill walking (Fitzsimmons et al., 2009; Foster et al., 2014). Behavioral monitoring and reward delivery are two important components for closed-loop behavior studies that are often absent in literature discussing wireless recording techniques

Studying behavior in primate models utilizes a variety of automated and manual methods. Joysticks and torque measuring systems record overt motor movements and can be used to trigger reward (Eaton et al., 2016) or to quantify behaviors related to neural activity (Fetz and Baker, 1973). Adapting these systems to a free behavior environment, such as the monkey's cage, is problematic. Some studies have elicited behaviors by manually presenting food rewards and using offline syncing methods to align the data (Jackson and Fetz, 2007; Fernandez-Leon et al., 2015). This strategy may work for short-term studies, but becomes unfeasible for long experimental sessions. Automated behavior tracking with invasive muscle activity sensing (Jackson and Fetz, 2007; Eaton et al., 2016) only provides information on recorded muscles. Traditional video tracking (Fitzsimmons et al., 2009; Foster et al., 2014; Schwarz et al., 2014) requires manually processing video frames or placing markers on the monkey (Vargas-Irwin et al., 2010) which typically deteriorate over long, unrestrained sessions. Tracking free behavior presents a trade-off between the quantity of information and the ability to process the data in real time. New advances in single camera depth tracking (Shotton et al., 2013) can provide easier, lower cost solutions to tracking behaviors.

Here we describe a novel system to enhance free-behavior experiments. Our approach allows for short-latency (<500 ms) reward delivery and behavior monitoring using off-the-shelf, low-cost components. Further, our method takes the first steps toward fully describing a monkey's natural behavior in its home environment, through an automated motion capture system. We have modified the Neurochip to wirelessly trigger a feeder to provide short-latency rewards contingent on the monkey's neural activity. Additionally, we have developed novel algorithms for monitoring the animal's behavior using the Microsoft Kinect, a motion tracking camera system. Finally we show examples of the systems working in tandem in a novel operant conditioning paradigm.

## Materials and methods

**Figure 1:**
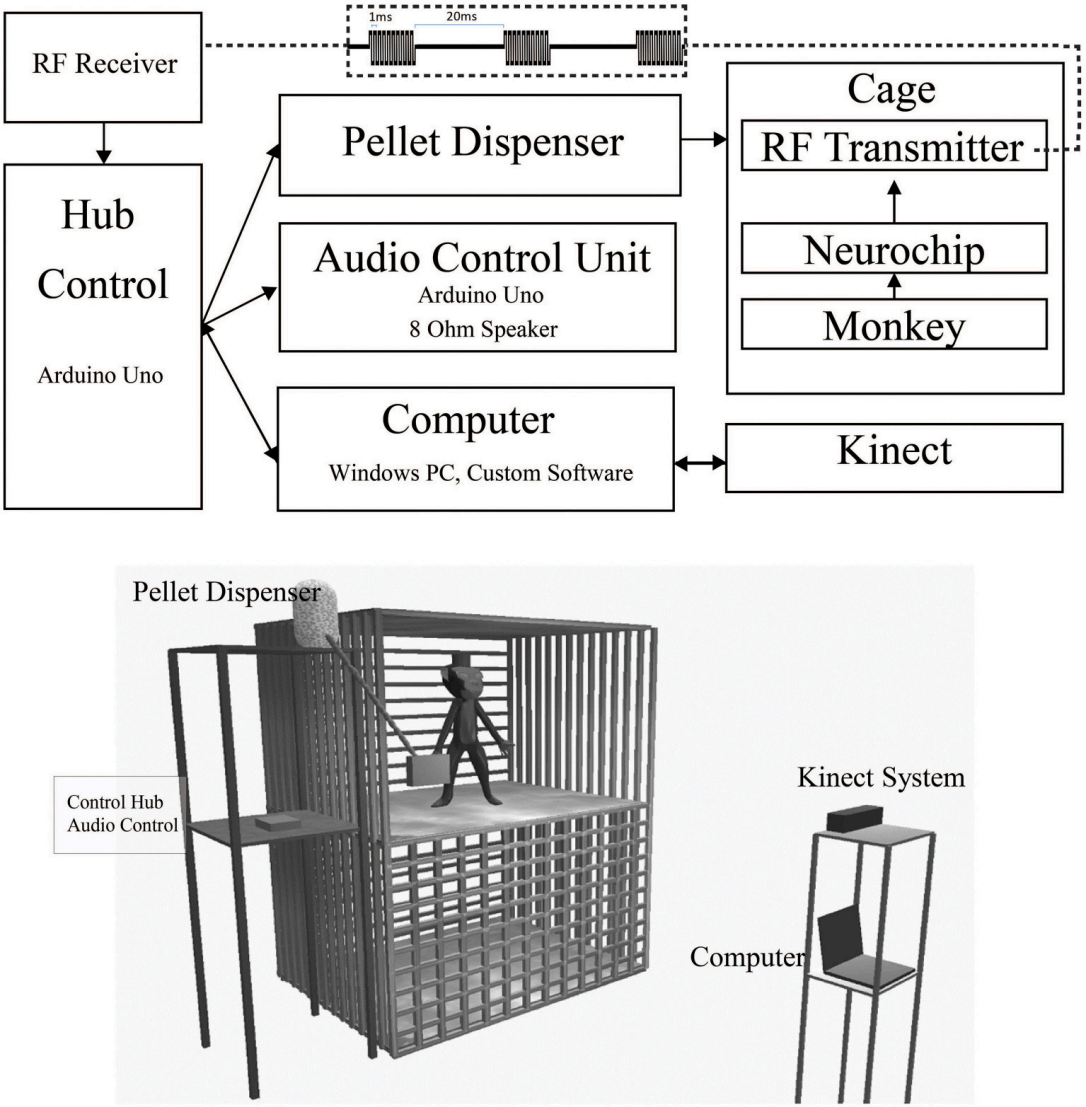
**Free-behavior hardware setup**. The overall system consists of off-the-shelf components and custom programmed control units. **Top**. Block Diagram of primary hardware components. Wired/wireless connections are indicated by solid/dashed lines. Bidirectional USB communication is indicated by double ended arrows. Wireless RF waveform is indicated by the top dashed inset. **Bottom**. In-cage system setup. The 3' × 3' × 3' cage is made of metal. The front bars in the top portion of the diagram have been removed to provide a clearer view of the monkey's environment. The control hub, audio, and pellet dispensing units were mounted on a stand next to the monkey's cage. The Kinect and computer were mounted on a separate stand outside the monkey's cage.

**Figure 2:**
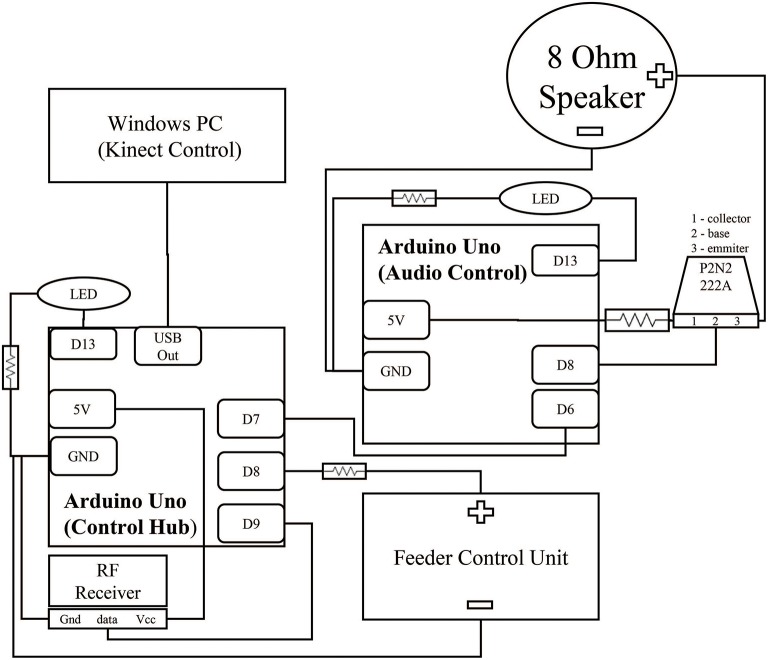
**Circuit for control hub and surrounding components**. Two Arduinos control the acquisition of the reward event and the subsequent audio feedback signal. Brown lines represent wiring between components. The black squares within the Arduinos represent individual connections to ports. Digital output ports (Dn) were selected for physical convenience and could be interchanged depending on experiment needs. Both Arduinos contain an LED link to extend the embedded Arduino LED feedback system from PIN 13 to increase visibility from further away. This LED was hidden from the monkey's view during experimentation. All hardware was packaged in a simple cardboard box.

Here we describe the individual components that allow untethered natural reinforcement of neural signals and the monitoring of associated behaviors. When a desired behavior is detected, the Neurochip provides an event signal via radio frequency (RF) transmission to a nearby RF receiver attached to an Arduino Uno control hub (Figure 1). This control hub relays this signal to an audio control unit, a pellet dispenser, and a Windows PC (Figure 2). The PC records data from a Microsoft Kinect sensor facing the monkey's cage. Software for the control units was written using the Aruidno IDE, and the PC was programmed in C#/Windows Presentation Format(WPF) using Visual Studio 2013. All software is available via a standard MIT License at http://depts.washington.edu/fetzweb/frontiers.html.

### Wireless communication from neurochip to control hub

The Neurochip has multiple auxiliary ports for connecting low-bit rate signals, such as an LED or small speaker. We connected a small, low-cost, 433 MHz RF transmitter (Model XY-FST FS1000A, JMoon Technologies) to this port allowing for wireless transmission of simple signals to an RF receiver (Model XY-MK-5V, JMoon Technologies) outside the cage. The Neurochip can pulse the auxiliary port at fixed intervals or in response to a detected event in the neural signal. Each transmission event consists of 3 sets of 10 biphasic pulses of 1 ms pulse width at 1000 pulses/s, repeated with a 20 ms delay between each set (Figure 1[top onset]). This signal is received by the RF receiver connected to an Arduino Uno, which processes the signal for controlling the feedback systems. This paradigm provides redundancy within the system, increasing security of event detection. This system enabled us to achieve over 99% efficiency, calculated by comparing recorded Neurochip events to transmitted events received by the control hub; only 1 or 2 events were missed during sessions lasting up to 10 h with hundreds of events. In our application, the signal triggered a feeder system attached to the side of the 3' × 3' × 3' cage, so our required range was less than five feet at all times, though the system could feasibly work for up to 10 feet.

### Reinforcement through food reward and audio feedback

The RF receiver is connected to an Arduino microcontroller hub (Control Hub), which sends control signals to three separate systems when an RF pulse is received:

1. A cage-mounted *feeder* that dispenses food pellets to the monkey. The feeder is positioned on the side of the monkey's cage with the dispensing tube leading to a small trough attached to the front of the cage.
2. An *audio control unit* consisting of an additional microcontroller to handle audio feedback. For each detected event, the audio control unit produces 3 short beeps to cue the animal that food reward is available and to also provide a secondary reinforcer. Constant tones can be used to distinguish between periods when food is available and when it is not. In control experiments the audio control unit can be configured to trigger additional long-term audio feedback to distinguish between control epochs. For example, a white noise can cue the monkey during rest periods. The pulse width of the feedback beeps and all audio frequencies can be easily configured with a minimum on time of 50 ms and a range of 10–4,000 Hz.
3. A connected *computer* to sync multiple streams of data. This computer handles the behavioral monitoring data streams and provides experiment updates via a Wi-Fi based notification system.

### Behavioral monitoring

**Figure 3:**
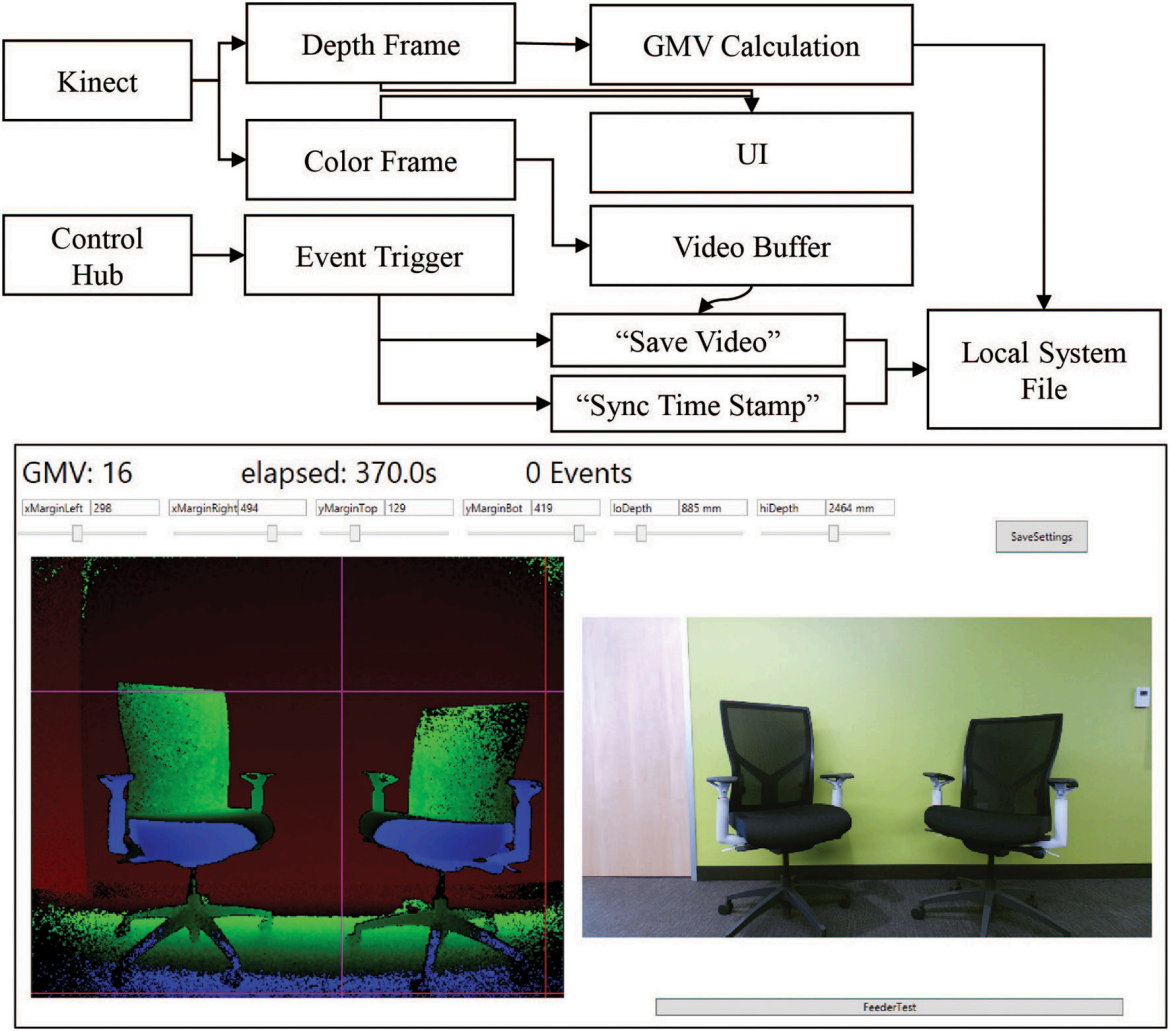
**Kinect software setup. Top**. Information flow block diagram. Behavioral data is collected using a combination of the Kinect data streams and the control hub event triggers. The Kinect produces simultaneous color (bottom right) and depth value (bottom left) frames of the scene. Both frames are sent to the UI for visualization of the scene to set margin and depth cutoff values. The depth frame is also passed to the calculation module of the main program. The color frame is passed to a video buffering module which maintains a constant 8-s video. When the control hub sends an event trigger to the program, the video buffer is allowed to run 4 additional seconds before saving the video. The event trigger also triggers a time stamp value to sync the movement calculation. The calculations and the videos are saved to file in real time to prevent excess memory requirements. **Bottom**. The primary UI of the system displays key elements for setting up the behavior monitoring system. The current GMV value, elapsed time and event count are displayed in the top left corner. The depth (left) and color (right) streams are displayed in real time. Each margin and depth cutoff setting has its own slider for on-the-fly adjustments of the field of view for the GMV calculation. Clicking “SaveSettings” applies the settings at the start of the next session. The feeder test button sends a test pulse to the control hub to trigger the feeder and the “Save Video Test” button triggers the video saving functioning of the program.

Our system combines event-triggered video and depth sensing to create an automated record of the animal's behavior during an experiment. A Microsoft Kinect camera measures the animal's physical location through a combination of traditional video and infrared depth sensing. The camera is controlled through a computer and custom software. The software contains controllers for the video and depth data streams, movement calculations, data saving, and receiving input from the Arduino control hub (Figure 3 Top). Both the video and depth streams are visible in the User Interface (UI) during setup. The camera and computer are placed on a stand ~3 feet away from the monkey's cage. Precision placement of the system is not required, as custom software can specify the camera's field of view (Figure 3 Bottom). The top, bottom, right, and left margins of the analysis area can be chosen such that the movement calculations occur only within the set margins. This is primarily used to outline the cage and exclude other cages and animals from the viewport. The front and back depth field cutoffs can also be modified directly, making values outside of these cutoffs fall to zero to increase the depth specificity of the system. These controls limit excess reflections from the back side of a cage and minimize the effects of people passing between the cage and camera. The system is further programmed to send automated experiment updates via an SMTP email client for remote monitoring of the experiment. Updates consist of a system status report, total event counts, and the timestamp of the most recent event. These metrics are useful in determining that the system is still operational and whether the monkey is still engaged in the task.

### Gross movement measurements

**Figure 4:**
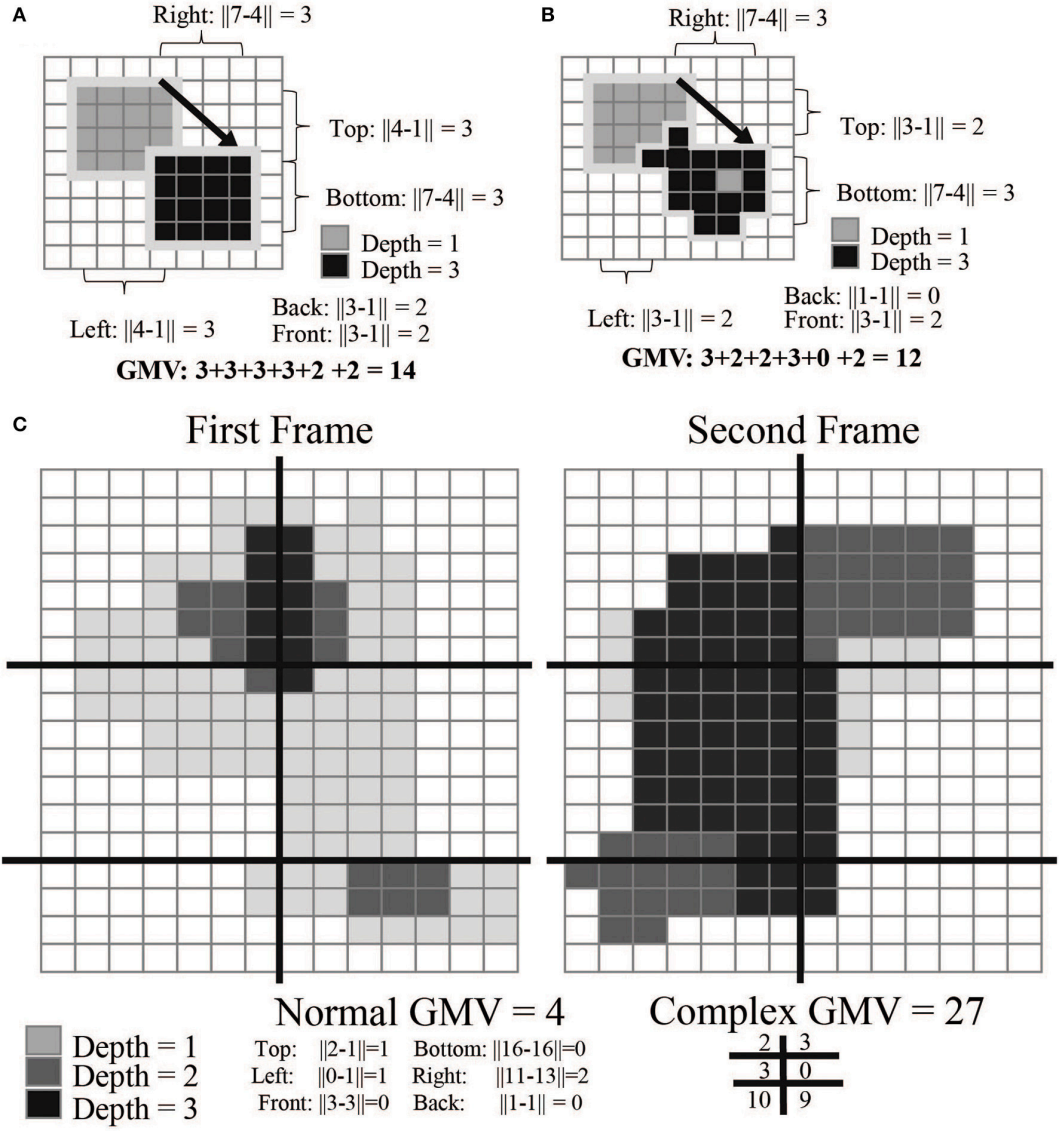
**GMV calculations**. The gross movement value (GMV) is calculated by detecting changes in pixels between frames. In examples **(A,B)**, the arrow indicates the transition from frame 1 to 2; example C is split into two panels. The absolute differences between the rightmost, leftmost, top-most, bottom-most, front-most, and back-most pixels are added together to calculate the final GMV. Example **(B)** is more complex than example **(A)**: the GMV for the top left is higher as more of the mass of the object has moved. Example **(C)** most closely mimics the calculation done while the monkey is in the cage environment. Each frame is broken down into segments (here represented as 6 different quadrants). By calculating the GMV per quadrant, we can detect regions of higher movement activity. This is useful in creating event-triggered averages of the GMV over the course of a full experimental session.

For compact movement monitoring we developed an algorithm that calculates an average movement value in user-specified regions of the camera's field of view. This “gross movement value” (GMV) is based on the movement of voxel values attributed to the animal; a high GMV indicates a large amount of movement. The GMV provides behavioral data throughout the free behavior experiment without any post-processing. This value primarily indicates the net amount of animal movement, but does not provide information on whether the animal was moving an arm vs. a leg. To calculate the value, the software finds the subject's topmost, bottommost, rightmost, leftmost, closest, and furthest voxels from the depth frame. It then compares these values to the previous frame to calculate a movement estimate (Figure 4).

$$\begin{array}{l} \text{GMV}(1)=||\text{Top}[1]-\text{Top}[0]||+||\text{Bottom}[1]-\text{Bottom}[0]||\\ \;+\;||\text{Right}[1]-\text{Right}[0]||+||\text{Left}[1]-\text{Left}[0]||\\ \;+\;||\text{ Front}[1]-\text{Front}[0]||+||\text{Back}[1]-\text{Back}[0]|| \end{array}$$

where 0 and 1 designate successive video frames. This value can be further refined by separating the camera's field of view into smaller quadrants. This total value can often reduce noise created by reflective elements in the camera's field of view (Figure 4C). The current GMV is displayed in the software UI in the top left corner.

$$\text{GMV}\_\text{total}={\text{GMV}}_{1,1}+{\text{GMV}}_{1,2}+.....{\text{GMV}}_{\text{n},\text{n}}$$

where subscripts denote the horizontal and vertical index of the quadrant.

The GMV has an inherent baseline that depends primarily on lighting and reflections of the scene. This is caused by the creation of the depth value frame which uses infrared light to illuminate the scene. If there are highly reflective objects (such as cage bars) the depth frame will flicker around those objects. This causes a baseline GMV that will be non-zero for most cases. In many applications, it is thus appropriate to normalize this value within a session. Reward events sent from the control hub are logged within the software and used to sync the GMV with the neural data offline.

### Event-triggered videos

Using the camera's color video feed, short video clips can also be recorded automatically through communication with the Arduino control hub. During an experiment the software continuously updates a buffer with recent video frames, configurable based on hardware. For our experiments we processed and saved every 4th frame at 32 fps. When the software receives a control signal from the hub, it triggers the software to save the previous frames as well as a set number of future frames, often totaling 4–8 s of video. This video snippet is then tagged with the event number and saved. This procedure creates short, easy to review videos during relevant time periods, such as reinforcement, circumventing the manual review required by traditional video monitoring.

### Operant conditioning of motor cortex field potentials during free behavior

This study was carried out in accordance with the recommendations of University of Washington Institutional Animal Care and Use Committee and all relevant regulatory standards. The protocol was approved by the University of Washington. One macaca nemestrina (Monkey J) was implanted (Eaton et al., 2016) with tungsten microwires (Jackson and Fetz, 2007) over the wrist area of motor cortex as part of a previous study (Eaton et al., 2016). Monkey J had been pre-trained on separate motor control tasks in both a traditional constrained environment and an in-cage environment (Eaton et al., 2016).

Monkey J was trained to volitionally modulate beta-band local field potentials (LFP) in the motor cortex while freely behaving in its cage. The Neurochip-2 system recorded these signals (digital bidirectional 3rd order bandpass 10–30 Hz) and calculated signal power from a running average of the rectified signal over a sliding 500 ms window. A threshold was determined using a 60-min baseline session during which no reward was given. A target threshold was set such that if the session had been a rewarded session, the monkey would have received a chosen number of rewards in the baseline period. This chosen reward number was varied over time to encourage better task performance but averaged around 1 reward per minute (1.02 ± 0.37, *n* = 14).

Increases in beta power above the target threshold caused the Neurochip to wirelessly trigger the control hub to deliver a food reward through the attached feeder. This was followed by a 5–10 s lockout period to prevent multiple rewards. This lockout was enforced by the Neurochip system. With each reward event the control hub triggered the motion capture system to save the event video and the audio feedback system to provide a reward tone. Food reward was available in 2–5 min epochs (Reinforced [R]), followed by 2–5 min non-rewarding epochs (Non-Reinforced [NR]). R periods were cued by an audible feedback tone located on the feeder (700 Hz) with each reward event triggering 3 beeps (1,200 Hz, 250 ms on, 250 ms off). Rewardable events were counted by the Neurochip during NR epochs for analysis purposes even though no food reward was delivered. The lockout protocols were present during the NR epochs for accurate comparisons.

Animal movement was characterized by the GMV. Since no NR events were transmitted to the feeder, triggers for analyzing GMV were not present during NR periods. This system allowed for a direct comparison between periods of volitional control and periods of rest, within the same experimental session. Control experiments, in which the feeder and tone were unavailable for long periods of time, and food was delivered at random intervals were used to document superstitious behavior. Through these experiments, we tested the efficacy of a totally wireless system in training a monkey to volitionally control brain activity.

## Results

### Effect of system on local field potential signals

**Figure 5:**
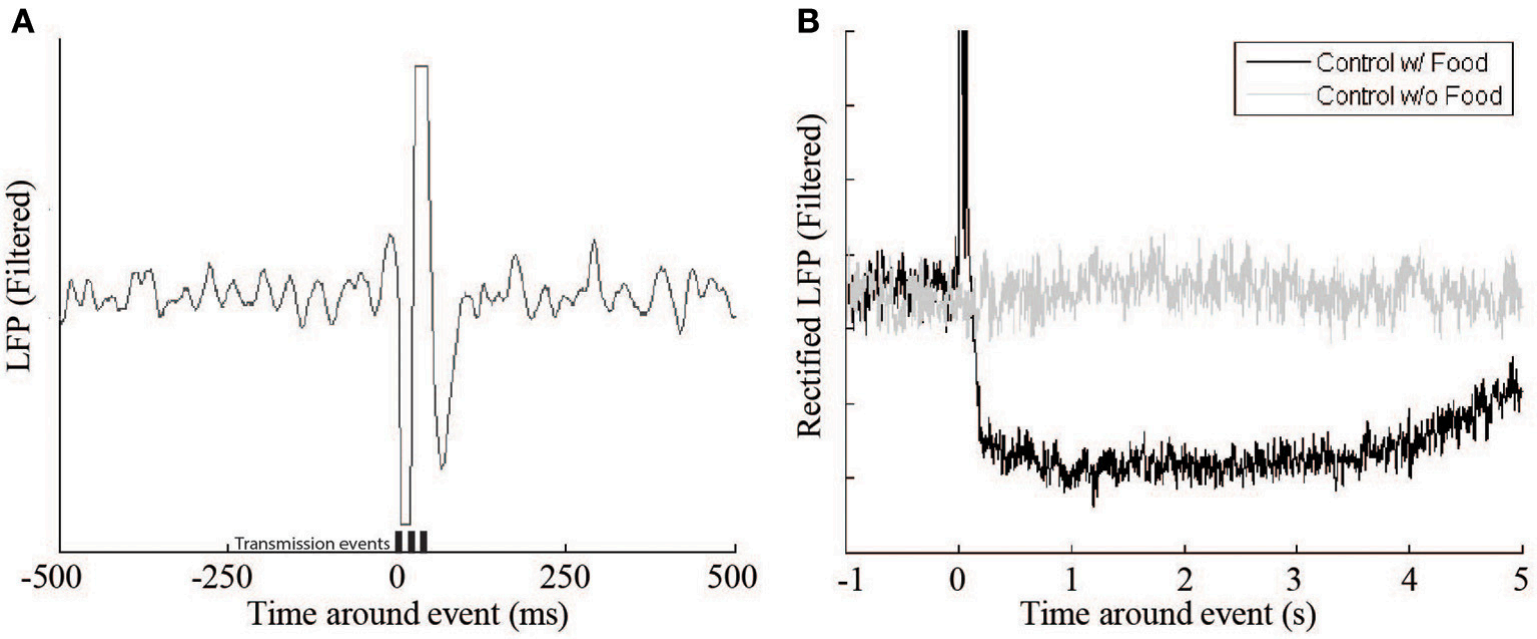
**Beta LFP signal response during control experiments. (A)** This figure shows a single Radio Frequency transmitter artifact during a control experiment where no food reward or feedback was delivered. Transmission events are represented by the bars (*t* = 0, 20, 40 ms). **(B)** The receipt of a food pellet (black trace) causes a drop in overall beta power compared to a non-food event (gray trace).

The system has multiple effects on the recording of the monkey's cortical activity. The first, and most apparent, is the signal artifact produced by the transmission of the RF signal (Figure 5 [left]). This artifact lasts 100–150 ms, which is slightly longer than the duration of the 60 ms signal transmission period, but has no other lasting effects on the underlying signal. Another important effect of the feeder system on the neural signal relates to the animal's behavior. When the animal retrieved the pellet from the hopper after delivery, there was a decrease in beta activity (Figure 5B). The signal returns to baseline after 3–10 s. To accommodate this pause a lock-out period was introduced in which no events could be triggered within 10 s of a previous event.

### GMV correlates negatively with signal power

**Figure 6:**
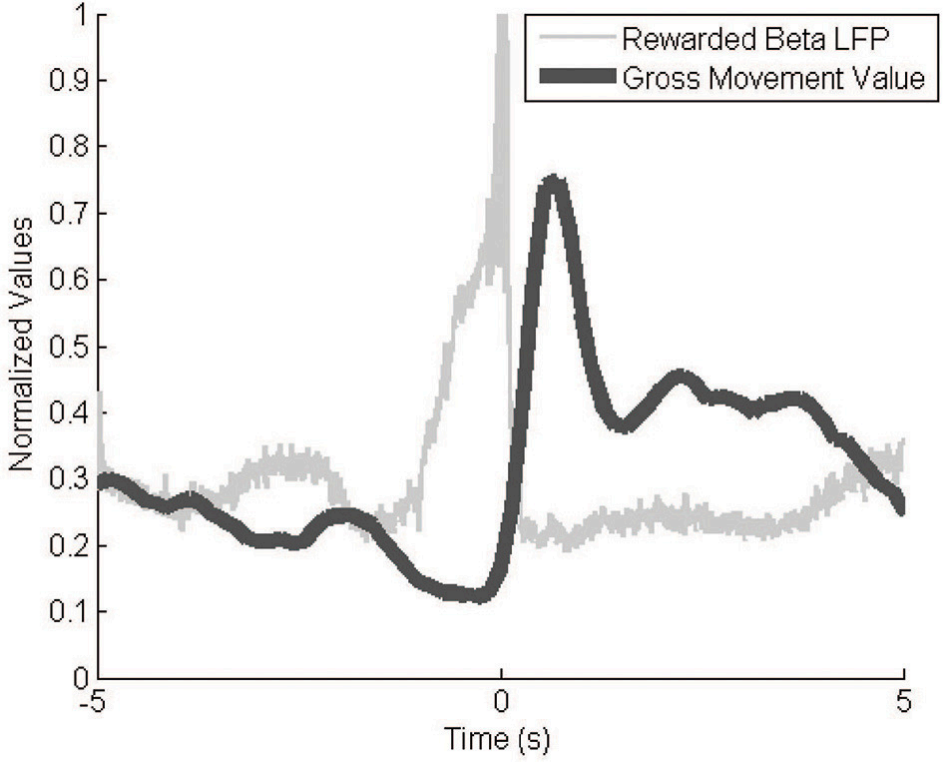
**GMV vs. LFP beta power**. The inverse relationship between signal power and the GMV is apparent during reward events. The filtered (10–30 Hz) and rectified neural signals and the GMV were averaged over 287 reward events while the animal was freely behaving in its cage. GMV is normalized to the maximum and minimum values from the entire session.

The Kinect behavior monitoring system recorded event-triggered videos and movement values as expected. Video represented only relevant behavioral data, a much smaller amount of data than continuous video. The GMV modulated greatly over the course of an experiment, with a typical dynamic range of 100. Baseline values varied slightly across experiments (±10 GMV) so we normalized the values to the within-session GMV range. The GMV correlated negatively with signal power (Figure 6). The video footage and GMV both show decreases in gross movement immediately prior to a reinforcement event. After the event, the GMV peaks as the monkey reaches to retrieve the pellet. This inverse correlation between movement and signal power in the 10–30 Hz range agrees with previous findings (Sanes and Donoghue, 1993).

### Volitional control of cortical signals is trainable during free behavior

**Figure 7:**
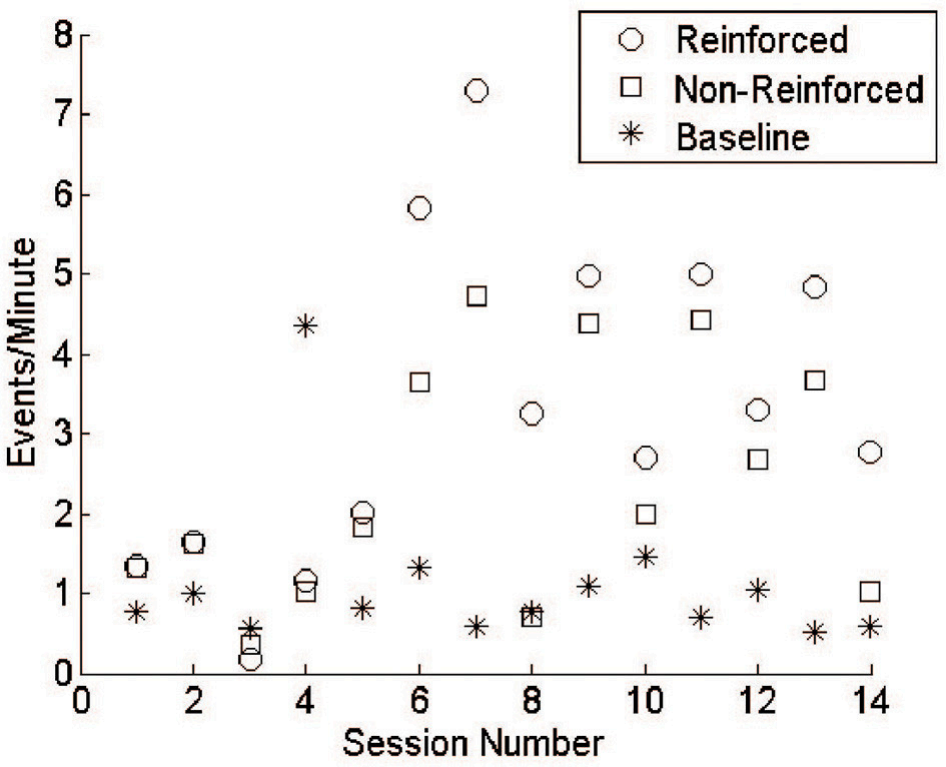
**LFP conditioning results in free behavior**. During early sessions reinforceable events during R and NR remained close to baseline responding. Events during R and NR increased above baseline by session 6 and fluctuated over the subsequent sessions. Training was not performed daily, as emphasis was given to system design and refinement.

Monkey J underwent 14 training sessions with LFP lasting 4.37 ± 0.64 h. Comparing the reward rate between R and NR epochs shows that, over time, the monkey was able to distinguish when reward was available and increase task performance accordingly (Figure 7). It is important to note, however, that the thresholds were not constant throughout these sessions, varying with daily baseline rates and experimental design. In most experiments, threshold was set to reward 1 event per minute, while still maintaining a whole-number value to compare against the integer-based power calculation. In session 4, however, the threshold was set incorrectly, potentially leading to worse behavior than would have occurred normally. A more rigorous training regimen would likely produce more consistent increases over time and a larger difference between R and NR periods. For these reasons the data were not subjected to statistical analysis, which would assume constant conditions over time.

**Figure 8:**
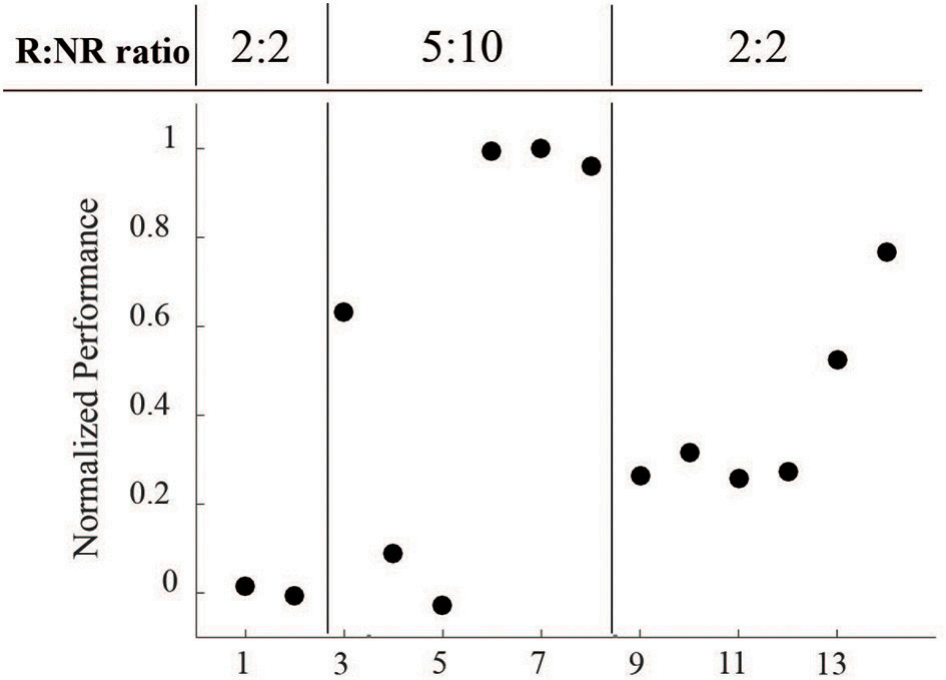
**Normalized performance across varying reinforced epoch ratios during LFP conditioning**. Performance, as measured by R events per minute minus NR events per minute, normalized to the maximum value, was directly affected at each epoch ratio transition, but returned to higher levels over time.

Another important consideration is that the length of R and NR epochs were modified throughout the course of these sessions (Figure 8). Sessions 1 and 2 started with 2 min R: 2 min NR ratio, which we hypothesized was too short an epoch to easily acquire the task. Sessions 3–8 were therefore increased to a 5 min R: 10 min NR ratio to allow for a longer learning and longer rest period. With the success of session 8, we returned the ratio to the starting 2:2 ratio to provide a more even comparison.

**Figure 9:**
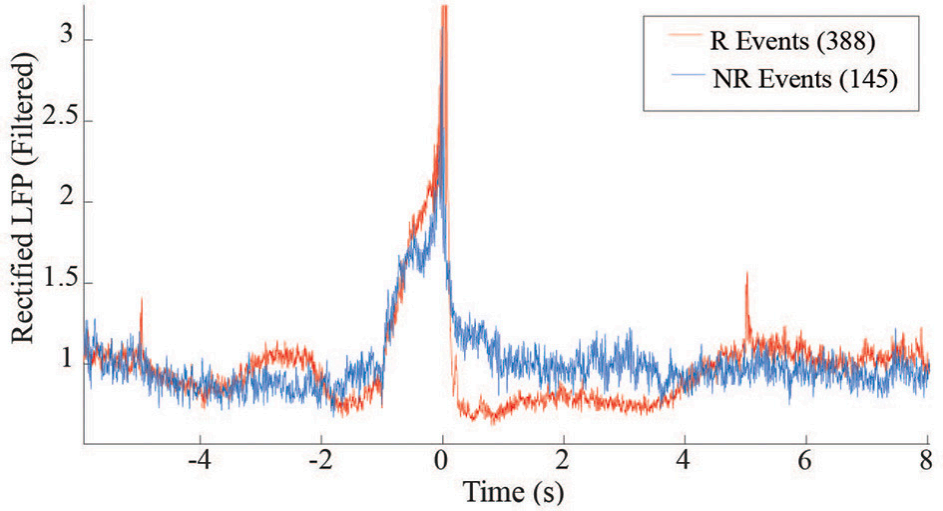
**LFP power differences between R and NR reward events in free behavior conditioning**. Band-passed (10–30 Hz) and rectified LFP signals were averaged across events occurring during R (red trace) and NR (blue trace) epochs.

The distribution of average rewardable events between R and NR epochs further demonstrate the monkey's ability to discriminate when reward was available, but more importantly indicates the lack of false positives that occurred during the NR periods. This distinction may reflect volitional actions toward retrieving the food pellets. Further insight into the difference between the R and NR periods can be obtained by comparing the average beta power around the triggered events (Figure 9). The post-reward drop in beta during R (red trace) is absent during NR (blue trace), when there is no pellet to retrieve.

At the beginning of each experiment, the threshold was set based on a 30-min baseline session during which no rewards were available. In all but one session, the rate of responding for both R and NR epochs was higher than during the baseline sessions (Figure 7). Though audio feedback was available, the high values during NR may reflect superstitious behavior. The last session (14) showed a clear difference between R and NR.

### Event-triggered videos show relevant behaviors

The event-triggered video system worked reliably in all 14 experimental sessions, producing 4-s video snippets showing the monkey's behavior in a clear and concise manner. There was no common pattern of monkey J's location or posture prior to a successful event. Some videos showed pacing behaviors while others showed the monkey sitting on one side of the cage. Almost all videos, however, show a slight pause in movement immediately before each event. This is consistent with the average GMV values which decreased prior to the event trigger (Figure 6). For the purpose of these proof-of-concept experiments, the event-triggered videos validate the GMV. In other experimental setups, they could be used to study more detailed nuances in behavior.

## Discussion

### Implications for BMIs

We present a novel system for studying operant conditioning paradigms and BMI systems in an untethered environment. We show an example of the system in use with an unrestricted monkey controlling specific brain signals to acquire rewards over multiple unsupervised hours. This, to our knowledge, is the first demonstration of a freely behaving BMI task with a purposeful end effector and behavioral monitoring. This implementation is significantly different from treadmill models that require specific behaviors, since our system allowed the animal to perform any behavior to accomplish the task.

This type of system has advantages for many novel experiments. Operant conditioning studies are possible over longer sessions while the animal is freely behaving, allowing ample time and freedom to discover the reinforced operant. Applications toward novel BMI control paradigms can be more easily explored in an automated way as these systems require limited personnel to operate. They can be adapted to run in parallel for multiple subjects. New on-chip signal processing algorithms and alternative neural signals and sites can be tested quickly, enabling the best solutions to be prioritized for further evaluation in human studies. Prime targets for this type of exploration include volitional control of prefrontal cortex neural signals(Kobayashi et al., 2010) and the use of new signal processing techniques (Bryan et al., 2013). The system removes traditional task and booth training from the experiment timeline These advantages will make it easier to study many new BMI systems under free conditions.

### Comparison to other systems

This paper presents an integrated system consisting of (1) an autonomous head-fixed brain-computer interface that wirelessly delivers activity-dependent feeder commands, (2) a cage-mounted feeder system with audio feedback, and (3) a behavior monitoring system. A range of commercial products are available that perform components of our overall system, but we are unaware of any single product that includes all of them. Each of these components can be interchanged with relevant available systems. Wireless recording systems, such as the Hermes line (Miranda et al., 2010) and others, could be outfitted with an RF transmitter. Commercial systems for wireless multichannel data transmission are available from various vendors, including Triangle Biosystems (http://www.trianglebiosystems.com), AD instruments (cdn.adinstruments.com), Ripple (http://rippleneuro.com), NeuraLynx (http://neuralynx.com) and Alpha Omega (https://alphaomega-eng.com). These could transmit data to an external computer programmed to detect neural activity of interest and deliver contingent feeder commands.

Several alternatives for reward systems exist. Commercial sources for feeders include Med Associates [http://www.med-associates.com], Lafayette Instrument Company [http://lafayetteneuroscience.com], Crist Instruments [http://www.cristinstrument.com]. The feeder system could be modified to provide additional secondary reinforcers, such as lights or audio feedback, using the Arduino system's open architecture.

For behavioral monitoring, investigational systems, such as the treadmill model (Foster et al., 2014), and commercial systems such as Clever Sys Inc.'s PrimateScan utilize multiple cameras spaced around the behavioral arena. These systems offer greater resolution for documenting behavior than our system. However, they require additional resources to operate that may increase the barrier to entry. Our system uses off-the-shelf components that are easy to acquire. Further, many of these systems require clear lines of sight to the subject, limiting their use in cages with bars between subject and cameras. The removal of cage bars clearly allows for better study of the monkey's behavior, but may not be feasible in many research environments. Behavioral monitoring with our system is useful for applications where precise monitoring of individual joint angles is not critical. Further, the GMV provides an immediately available behavioral data stream without manual processing of video frames. While frame-by-frame analysis can yield higher resolution, it may not be feasible for prolonged experiments. Our described system is ideal for preliminary studies where a gross movement measurement could be used to justify and inform further study with more expensive equipment.

### System costs

The system we have described is highly modular. Any given component could be replaced by a similar piece of hardware, depending on the needs of a specific experiment. With the exception of the Neurochip and pellet dispenser system, the total build cost was ~$250 (Table 1). Neurochips can be made available for purchase upon request to author E. Fetz. The latest version of the Neurochip includes 16 (dual input) or 32 (single-ended input) recording channels (Intan amplifier), 3-axis accelerometer, programmable Altera CycloneV FPGA and Atmel SAM4 CPU, 6 high-compliance stimulating channels and 64–128 GB on-board data storage. The cost of this “Neurochip3” system is about $5,000, depending on configuration. The cost of commercial products performing these functions depends on specific system specs, but the total will exceed that of the overall system described here. For details on particular commercial options the reader is referred to the vendors' websites, where current prices can be requested.

**Table 1:** **Approximate hardware costs**.

2 x Arduino Uno	$80	Arduino
Kinect	$120	Microsoft
RF transmitter (XY-FST FS1000A) and RF receiver (XY-MK-5V) package	$5	JMoon Technologies
Stand equipment (PVC pipe, plastic sheeting, Zip ties)	$30	Home Depot
8 Ohm speaker (CLS0261MAE-L152)	$5	Digi-Key
Wire, resistors, transistors	$10 (est.)	In-house

### Reward latency

The delay between reward event and pellet delivery was difficult to measure accurately but was less than 500 ms. This was estimated by watching an LED on the Neurochip turn on after a successful reward event and hearing the pellet drop into the pellet retrieval trough. A more robust quantification would be possible with additional sensors, such as an infrared interrupter switch attached to the Arduino control hub. This latency is primarily the time required for the pellet to travel down the dispensing tube and could be reduced by making the dispensing path shorter. The larger reward latency comes from the monkey retrieving the pellet from the pellet trough, which was placed on the outside of the cage to prevent damage; future iterations could place this trough inside the cage to shorten retrieval time.

### External distractions

One of the main drawbacks to this system, and free-behavior systems in general, is the lack of control over the exterior environment. In our setup, the animal was returned to its cage in a room with other monkey cages. Activity of other monkeys or personnel may be a distraction that affects the “natural” behavior of the animal. Overall, these effects were minor in our testing, but should be considered when planning behavioral experiments.

### Improvements

Further improvements to the transmission and receiving protocols could increase the range and efficiency of the system. RF transmission is cheaper than Bluetooth and smaller than most Wi-Fi modules, but lacks the range and efficiency of these alternatives. Our transmission and receiving protocols produce very few dropped events; however dropped events could increase in some circumstances, such as when the animal was hunched over away from the receiver. This could be remedied by placing multiple receivers around the cage, at the cost of increased setup complexity. Additionally, the current protocols need to be modified if multiple systems are operating in the same room. Adding a unique ID code to the RF transmission signal could provide one solution, but could also decrease the chance of detecting an event. New, low-cost options in Bluetooth and Wi-Fi could address these issues.

Improvements in motion tracking could aid in creating a more precise movement value that can categorize different types of movements. The second generation of Kinect can provide more accurate readings of the depth values at higher resolutions. This should allow more accurate documentation of kinematics in real time, including joint angles and monkey orientation.

## Conclusions

We have developed the first comprehensive system for rewarding untethered monkeys during free behavior through a cage-mounted feeder system. This system was controlled wirelessly through a small adapter to the Neurochip 2 system, enabling wireless reinforcement of neural modulations. The system was paired with a novel motion capture system that documented relevant behavioral data and event-related video snippets. The system exhibits reward delivery latencies (<500 ms) comparable to training-booth food-delivery systems. Initial experiments conducted with the feeder have shown promising behavioral results in training a monkey to modulate LFP during free behavior. Future refinements to the radio frequency communications protocol and microcontroller programming will enable greater transmission distances and simultaneous operation of multiple systems.

## Author contributions

TL and EF conceived the experiments; TL performed the experiments and TL and EF wrote the manuscript.

## Funding

This work was supported by NSF DGE-1256082, the NSF ERC Center for Sensorimotor Neural Engineering and NIH RO1 NS 12542 and RR 00166.

### Conflict of interest statement

The authors declare that the research was conducted in the absence of any commercial or financial relationships that could be construed as a potential conflict of interest.

## References

[B1] Aflalo T. N. Graziano S. M. (2006). Partial tuning of motor cortex neurons to final posture in a free-moving paradigm. Proc. Natl. Acad. Sci. U.S.A. 103, 2909–2914. 10.1073/pnas.0511139103 16473936 PMC1413833

[B2] Anderson D. M. (2008). The nonhuman primate as a model for biomedical research, in Sourcebook of Models for Biomedical Reasearch, ed Conn P. M. (Totowa, N. J: Humana), 251–258.

[B3] Bryan M. J. Martin S. A. Cheung W. Rao R. P. (2013). Probabilistic co-adaptive brain-computer interfacing. J. Neural Eng. 10:066008 10.1088/1741-2560/10/6/066008 24140680

[B4] Caminiti R. Johnson P. B. Burnod Y. Galli C. Ferraina S. (1990). Shift of preferred directions of premotor cortical cells with arm movements performed across the workspace. Exp. Brain Res. 83, 228–232. 10.1007/BF00232214 2073945

[B5] Chen J. Reitzen S. D. Kohlenstein J. B. Gardner E. P. (2009). Neural representation of hand kinematics during prehension in posterior parietal cortex of the macaque monkey. J. Neurophysiol. 102, 3310–3328. 10.1152/jn.90942.2008 19793876 PMC2804418

[B6] Eaton R. Libey T. Fetz E. E. (2016). Operant conditioning of neural activity in freely behaving monkeys with intracranial reinforcement. J. Neurophysiol. 17, 1112–1125. 10.1152/jn.00423.2016 PMC5340878 28031396

[B7] Fernandez-Leon J. A. Parajuli A. Franklin R. Sorenson M. Felleman D. J. Hansen B. J.. (2015). A wireless transmission neural interface system for unconstrained non-human primates. J. Neural Eng. 12:056005. 10.1088/1741-2560/12/5/056005 26269496 PMC5996767

[B8] Fetz E. E. Baker M. A. (1973). Operantly conditioned patterns on precentral unit activity and correlated responses in adjacent cells and contralateral muscles. J. Neurophysiol. 36, 179–204. 4196269 10.1152/jn.1973.36.2.179

[B9] Fetz E. E. Finocchio D. V. (1975). Correlations between activity of motor cortex cells and arm muscles during operantly conditioned response patterns. Exp. Brain Res. 23, 217–240. 10.1007/BF00239736 810359

[B10] Fitzsimmons N. A. Lebedev M. A. Peikon I. D. Nicolelis M. A. L. (2009). Extracting kinematic parameters for monkey bipedal walking from cortical neuronal ensemble activity. Front. Integr. Neurosci. 3:3. 10.3389/neuro.07.003.2009 19404411 PMC2659168

[B11] Foster J. D. Nuyujukian P. Freifeld O. Gao H. Walker R. I'Ryu S.. (2014). A freely-moving monkey treadmill model. J. Neural Eng. 11:046020. 10.1088/1741-2560/11/4/046020 24995476

[B12] Griffin D. M. Hudson H. M. Belhaj-Saïf A. McKiernan B. J. Cheney P. D. (2008). Do corticomotoneuronal cells predict target muscle EMG activity? J. Neurophysiol. 99, 1169–1986. 10.1152/jn.00906.2007 18160426

[B13] Hochberg L. R. Bacher D. Jarosiewicz B. Masse N. Y. Simeral J. D. Vogel J.. (2012). Reach and grasp by people with tetraplegia using a neurally controlled robotic arm. Nature 485, 372–375. 10.1038/nature11076 22596161 PMC3640850

[B14] Ifft P. J. Lebedev M. A. Nicolelis M. A. (2012). Reprogramming movements: extraction of motor intentions from cortical ensemble activity when movement goals change. Front. Neuroeng. 5:16. 10.3389/fneng.2012.00016 22826698 PMC3399119

[B15] Jackson A. Fetz E. E. (2007). Compact movable microwire array for long-term chronic unit recording in cerebral cortex of primates. J. Neurophysiol. 98, 3109–3118. 10.1152/jn.00569.2007 17855584

[B16] Jackson A. Mavoori J. Fetz E. E. (2006a). Long-term motor cortex plasticity induced by an electronic neural implant. Nature 444, 56–60. 10.1038/nature05226 17057705

[B17] Jackson A. Mavoori J. Fetz E. E. (2007). Correlations between the same motor cortex cells and arm muscles during a trained task, free behavior, and natural sleep in the macaque monkey. J. Neurophysiol. 97, 360–374. 10.1152/jn.00710.2006 17021028

[B18] Jackson A. Moritz C. T. Mavoori J. Lucas T. H. Fetz E. E. (2006b). The Neurochip BCI: towards a neural prosthesis for upper limb function. IEEE Trans. Neural Syst. Rehabil. Eng. 14, 187–190. 10.1109/TNSRE.2006.875547 16792290

[B19] Kobayashi S. Schultz W. Sakagami M. (2010). Operant conditioning of primate prefrontal neurons. J. Neurophysiol. 103, 1843–1855. 10.1152/jn.00173.2009 20107129 PMC2853276

[B20] Miranda H. Gilja V. Chestek C. A. Shenoy K. V. Meng T. H. (2010). HermesD: a high-rate long-range wireless transmission system for simultaneous multichannel neural recording applications. IEEE Trans. Biomed. Circuits Syst. 4, 181–191. 10.1109/TBCAS.2010.2044573 23853342

[B21] Moritz C. T. Fetz E. E. (2011). Volitional control of single cortical neurons in a brain-machine interface. J. Neural Eng. 8:025017. 10.1088/1741-2560/8/2/025017 21436531 PMC3156089

[B22] Sanes J. N. Donoghue J. P. (1993). Oscillations in local field potentials of the primate motor cortex during voluntary movement. Proc. Natl. Acad. Sci. U.S.A. 90, 4470–4474. 10.1073/pnas.90.10.4470 8506287 PMC46533

[B23] Santhanam G. Ryu S. I. Yu B. M. Afshar A. Shenoy K. V. (2006). A high-performance brain-computer interface. Nature 442, 195–198. 10.1038/nature04968 16838020

[B24] Schwarz D. A. Lebedev M. A. Hanson T. L. Dimitrov D. F. Lehew G. Meloy J.. (2014). Chronic, wireless recordings of large-scale brain activity in freely moving rhesus monkeys. Nat. Methods 11, 670–676. 10.1038/nmeth.2936 24776634 PMC4161037

[B25] Shotton J. Girshick R. Fitzgibbon A. Sharp T. Cook M. Finocchio M.. (2013). Efficient human pose estimation from single depth images. IEEE Trans Pattern Anal. Mach. Intell. 35, 2821–2840. 10.1109/TPAMI.2012.241 24136424

[B26] Taylor D. M. Tillery S. I. Schwartz A. B. (2002). Direct cortical control of 3D neuroprosthetic devices. Science 296, 1829–1832. 10.1126/science.1070291 12052948

[B27] Vargas-Irwin C. E. Shakhnarovich G. Yadollahpour P. Mislow J. M. K. Black M. J. Donoghue J. P.. (2010). Decoding complete reach and grasp actions from local primary motor cortex populations. J. Neurosci. 30, 9659–9669. 10.1523/JNEUROSCI.5443-09.2010 20660249 PMC2921895

[B28] Wander J. D. Collinger J. L. Degenhart A. D. Tyler-Kabara E. C. Schwartz A. B. Moran D. W.. (2013). Distributed cortical adaptation during learning of a brain-computer interface task. Proc. Natl. Acad. Sci. U.S.A. 110, 10818–10823. 10.1073/pnas.1221127110 23754426 PMC3696802

[B29] Wang W. Collinger J. L. Degenhart A. D. Tyler-Kabara E. C. Schwartz A. B. Moran D. W.. (2013). An electrocorticographic brain interface in an individual with tetraplegia. PLoS ONE 8:e55344. 10.1371/journal.pone.0055344 23405137 PMC3566209

[B30] Zanos S. Richardson A. G. Shupe L. Miles F. P. Fetz E. E. (2011). The Neurochip-2: an autonomous head-fixed computer for recording and stimulating in freely behaving monkeys. IEEE Trans. Neural Syst. Rehabil. Eng. 19, 427–435. 10.1109/TNSRE.2011.2158007 21632309 PMC3159515

